# Why language really is not a communication system: a cognitive view of language evolution

**DOI:** 10.3389/fpsyg.2015.01434

**Published:** 2015-09-24

**Authors:** Anne C. Reboul

**Affiliations:** CNRS UMR 5304, Laboratory on Language, Brain and Cognition (L2C2), Institute for Cognitive Sciences-Marc JeannerodBron, France

**Keywords:** language evolution, language-ready brain, communication, code model, ostensive model, Language of Thought, globularity

## Abstract

While most evolutionary scenarios for language see it as a communication system with consequences on the language-ready brain, there are major difficulties for such a view. First, language has a core combination of features—semanticity, discrete infinity, and decoupling—that makes it unique among communication systems and that raise deep problems for the view that it evolved for communication. Second, extant models of communication systems—the code model of communication (Millikan, 2005) and the ostensive model of communication (Scott-Phillips, 2015) cannot account for language evolution. I propose an alternative view, according to which language first evolved as a cognitive tool, following Fodor’s (1975, 2008) Language of Thought Hypothesis, and was then exapted (externalized) for communication. On this view, a language-ready brain is a brain profoundly reorganized in terms of connectivity, allowing the human conceptual system to emerge, triggering the emergence of syntax. Language as used in communication inherited its core combination of features from the Language of Thought.

## Introduction

Language evolution has been mainly approached through the evolutionary notion of *function*. As language is routinely used in human communication, the natural assumption is that the function of language is communication. As a consequence, theories of language evolution have centered on scenarios that try to explain the kinds of selection pressures that could have triggered the emergence of this rather remarkable communication system. Inevitably given that communication is the epitome of a social phenomenon, these scenarios have been “social”^1^. However, seeing language as a system of communication and proposing that it has evolved *as* a system of communication (i.e., seeing language as being a system of communication in the *strong* sense) rather than *being merely used in communication* (i.e., seeing it as being a system of communication in the *weak* sense) raises a host of difficult issues which have to do with the very nature of language. The question of whether language is or is not a communication system in the strong sense that it evolved *for* communication is far from anecdotal as its answer strongly constrains what a language-ready brain would comprise in terms of necessary preliminary cognitive abilities.

That language is eccentric among animal communication systems cannot be seriously disputed. It has a core combination of features—semanticity, discrete infinity, and decoupling—that is found nowhere else in nature to our present knowledge (Chomsky, 1966/2009). Relative to the evolution of language as a system of communication, this core combination of features raises two major difficulties:

(a) Given that it gives rise to linguistic creativity (the potential production of an infinite number of different sentences with different contents), it must be explained why humans—and only humans—need to be able to communicate a potential infinity of different contents;
(b) We are also owed an explanation of how, given that decoupling facilitates cheating and deceiving, while the evolution of communication systems is subject to stringent constraints of honesty (see Animal Communication Systems), a communication system that incorporates decoupling could ever get off the ground.

As Számado and Szathmáry (2006) have noted, none of the extant scenarios can satisfactorily answer these two questions^2^. Given these possibly intractable difficulties, it makes sense to reexamine the evidence in favor of the conclusion that language is a communication system and that it has evolved *as* a communication system.

Thus, the main goal of this paper is to assess the notion that language is a communication system in the strong sense. Here, a few words (for a complete presentation, see Animal Communication Systems) about what a communication system is are in order. The traditional view of communication systems is the code model^3^: the communicator encodes the message she wants to communicate, this encoded message is relayed along a channel to the receiver who decodes it and recovers the intended message. Though it is generally considered that this applies fairly well to animal communication systems^4^ (see Animal Communication Systems), there are serious doubts that it can apply to the use of language in human communication. This is because, as has been abundantly argued (Sperber and Wilson, 1995; Carston, 2002; Recanati, 2004, 2010, following in the steps of Grice, 1989), on the whole, the semantic meaning of an utterance (the *sentence meaning*) fails to correspond exactly to what the speaker intended to communicate (the *speaker’s meaning*). In other words, encoding–decoding processes are not sufficient to recover the message. While this *contextualist* position is by now largely acknowledged in both philosophy of language and linguistics, it did not penetrate the field of language evolution until very recently, when Scott-Phillips (2015) proposed a new view of language evolution. According to him, there are two main roads to the evolution of a system of communication:

(a) Almost all communication systems (i.e., animal communication systems) are congruent with a description in terms of the code model of communication and have evolved, independently of any social abilities, either biologically or culturally, signal by signal;
(b) A single other communication system (i.e., language) is an *ostensive* communication system^5^, and developed *as a system on top of an ostensive-inferential communicative mechanism* (based on the social ability of mind reading) through the establishment of a set of linguistic conventions.

In other words, while, on the code model view of language, it is *continuous with all other animal communication systems*, on the ostensive view of language, it is *discontinuous with all other animal communication systems*.

Obviously, arguments against the code model view of language as a communication system may well be inoperative against the ostensive view of language as a communication system. Thus, both theoretical frameworks will have to be examined, and we will begin with the most popular one, i.e., the code model.

## Language as a Communication System under the Code Model

As we have just seen, under the code model of communication, language is continuous with animal communication systems, and here it is useful to make a brief incursion into animal communication systems.

### Animal Communication Systems

Though whole books have been written on the subject of the evolution of communication in animals (e.g., Hauser, 1996; Oller and Griebel, 2004), their authors have often been content to use the word without giving it a precise definition. They rely on its vernacular meaning and on a rather vague notion of *information transfer*^6^, waving at Shannon and Weaver’s (1949) quantitative definition of *information*. As pointed out by Owren et al. (2010), this is usually accompanied by the idea that this transfer of information is based on an *encoding* (on the signaler’s side) and a *decoding* (on the receiver’s side) process^7^. It is this view of communication as information transfer that makes honesty central to the evolution of communication systems.

Another line of thought was opened by Krebs and Dawkins (1984), who claim that the root of the evolution of animal communication lies in manipulation, linking the sending of a signal (the unit of animal communication systems) to a response (by the recipient) advantageous to the signaler. This view of communication was clearly influential, as shown by Maynard Maynard Smith and Harper (2003, p. 3) definition of a signal:

“We define a ‘signal’ as any act or structure which alters the behaviour of other organisms, which evolved because of that effect, and which is effective because the receiver’s response has also evolved.”

In other words, the evolution of communication is not the evolution of the signal in isolation, but rather of pairs of signal–responses. This might be thought to go all the way toward a manipulation account of communication, but this is not the case. In the comments that follow, Maynard Smith and Harper outline some consequences of their definition that put them squarely on the information transfer side. First, if the signal affects the receiver’s behavior, it must do so in a way that is not, on the whole, detrimental to the receiver (otherwise selection would rapidly eliminate receptivity to it). Second, this means, on Maynard Smith and Harper’s view, that the signal must *reliably and honestly* (truthfully) convey information about the environment or about the signaler’s present state and/or future behavior. In other words, the signal evolved for its behavior-altering effects, but that does not mean that it does not carry information.

A stronger challenge to the information-based studies of communication has developed, however, through a series of papers by Owren et al. (2010, for a synthetic presentation), initially inspired by Krebs and Dawkins (1984), but presenting an alternative, rather than a mere addition to the information-based view. Owren et al.’s (2010) most convincing examples are mating signals. While mating signals have generally been analyzed in the information-based literature as transmitting information to females about males’ genetic worth^8^, Owren et al. (2010) propose an alternative view. Mating signals, whether visual, auditory, etc., are (in general) not informing the receiver of the signaler’s genetic quality, nor is it their function to do so. Rather, mating signals exploit pre-existing sensory preferences of females. These preferences usually have evolved in entirely different contexts (e.g., foraging for food), but once evolved they are ripe for exploitation. Thus, mating signs directly impinge on females’ sensory systems, and did not evolve for the purpose of transmitting (reliable) information about the signaler’s genetic value. It is important to note that Owren et al. (2010) do not exclude the possibility that mating signals may occasionally carry (reliable) information about the signaler’s genetic worth. Rather, if they do so, this is incidental. Their main function, which explains why they evolved, is not to signal fitness, but to attract females. This, basically, is Owren et al.’s (2010) alternative view of animal communication: its main function is not to transfer information between organisms, but to induce behaviors in the receiver that are advantageous to the communicator. Eschewing the negatively loaded term *manipulation*, they propose an *influence*-based view of animal communication.

This is clearly not the place to settle that debate (the interested reader is directed to the papers in Stegmann, 2013), but there one thing worth pointing out. While Owren et al. (2010) rightly deplore the detrimental effect on the animal communication literature of the (language-inspired) information-based approach, one may equally deplore the effects on the language evolution literature of an approach based on animal communication^9^, however, tainted by (mis-)conceptions of human language.

One of the best examples of a view of language evolution that sees language as continuous with animal communication systems, in keeping with the code model, is Millikan’s account of language and its evolution. I will mainly discuss her most recent book centering on language (Millikan, 2005).

### Millikan’s Account

Millikan’s approach to language belongs to the presently influential philosophical program aiming at “naturalizing” the mental^10^, concerning both mental representations and their communicative counterparts. In a move that has become classical in such programs, she aims at establishing a continuity between *natural* signs or meaning and *non-natural* signs or meaning.

The distinction between some form of natural signification (based on correlations that are, more often than not, grounded in causality) and linguistic signification is far from new, but it was given a paramount importance in Grice’s (1989) classical analysis of meaning, which is also Millikan’s main target. Grice’s strategy was to look at two uses of the verb *to mean*. Thus, he began by comparing the following examples:

(1) These spots mean (meant) measles.
(2) These three rings on the bus bell mean (meant) that the bus is full.

While in the first example, the verb *to mean* is used in its *natural* sense, in the second, it is used in its *non-natural* sense. Grice noted that these two uses of the verb are distinguished by the implications that one is entitled to draw from each of them. While natural meaning is *factive*, in the sense that *x means (meant) p* entails *p*, non-natural meaning (henceforth *meaning_nn_*) is *non-factive* in the sense that *x means_nn_ p* does not entail *p.* On the other hand, meaning_nn_ is *under voluntary control* in the sense that from *x means_nn_ p* one can deduce that *Someone meant_nn_ p by x*. However, natural meaning is *not under voluntary control* (it does not license the corresponding inference). So, in short, natural meaning is factive and not under voluntary control while meaning_nn_ is non-factive and under voluntary control.

Grice (1989, p. 219) went further, however, and added the following definition of meaning_nn_:

“A meant_nn_ something by *x*” is roughly equivalent to “A intended the utterance of *x* to produce some effect in an audience by means of the recognition of that intention.”

In other words, meaning_nn_ is not only under voluntary control: additionally, the speaker has a double intention:

• The *primary* intention to produce a given effect in her audience;
• The *secondary* intention to produce that effect *via* the audience’s recognition of her (the speaker’s) primary intention.

Grice was at pain to emphasize that the primary intention is crucial to the definition: cases where the audience recognizes the meaning without recognizing the primary intention are not cases of meaning_nn_. Additionally, Grice insisted that, though meaning_nn_ could be conventional, it did not have to be conventional. In other words, on Grice’s view, normal linguistic communication is not a matter of encoding and decoding as such, but rather of recognizing the speaker’s primary intention.

Grice’s account of meaning_nn_ has been Millikan’s target all along her philosophical career (the first instance was Millikan, 1984). Her goal has been to show that the psychological side of Gricean meaning_nn_ is not necessary, that linguistic communication is indeed a matter of encoding–decoding and that meaning is conventional in a utterly non-psychological sense^11^. In other words, what distinguishes natural signs or meaning from non-natural signs or meaning is only factivity, not volition: natural signs are factive, non-natural signs are not (the signaler may be mistaken or deceptive). Thus, Millikan’s distinction between natural and non-natural meaning is wholly non-psychological.

Millikan’s account of meaning centrally uses the notion of *function*, explicitly borrowed from evolutionary biology. Millikan (1984) introduces the notion of *proper function*, which is fundamentally *historical* in the following sense: it does not refers to what an entity (be it an organ or a behavior) actually does, but rather to why that entity not only exists now, but has persisted (possibly with modifications) since its emergence, in other words, why it has been selected for. So whatever the state of your heart, and regardless of whether it actually reliably pumps blood throughout your body, its proper function is to pump blood, because this is the reason why hearts have evolved, been preserved (and improved) throughout vertebrate history. Note that proper functions are not limited to biological organisms: they can also characterize artifacts of all kinds, from institutions to tools. In other words, they can be the product of either biological or cultural evolution. The essential thing is that the entity considered has a history which explains why it persisted throughout time by the function it normally performs.

On Millikan’s view, language is a communication system, on a par with the other animal communication systems, as far as its evolution is concerned. She shares with Maynard Smith and Harper’s (2003) definition of a signal the idea that signals evolve in tandem with responses (indeed, she views language as the solution to coordination problems in humans^12^). Her idea is that the proper function of a signal is to evoke a specific response in the receiver, and that it does so through information transfer^13^. While clearly the notion of information transfer involved applies to natural language as well as to animal communication systems, Millikan acknowledges that linguistic signals and animal signals are different up to a point. This can be seen through her analysis (Millikan, 2004) of vervet alarm calls. In linguistic terms, such calls (e.g., the leopard call) have a double direction of fit: both world-to-signal (i.e., the call reflects the current state of the environment, e.g., the presence of a leopard in it) and signal-to-world (i.e., the signal simultaneously enjoins the recipient to give a specific response, e.g., flying to the top of the canopy). Millikan proposes to call such double-directed signals *pushmi-pullyu representations*. As Millikan (2013) herself concedes, it does not make any sense to “translate” animal signals into language. For instance, the vervet leopard alarm call is in no way equivalent to the complex sentence “There is a leopard here and you must climb to the top of the nearest tree”. Though this might reflect fairly faithfully the meaning of the call, it is not a translation, because animal signals are, on the whole, holistic^14^: *the signal means something as a whole, not as a combination of its parts*. Indeed, as Millikan acknowledges, it is only with language that the two directions of fit (*indication* = world-to-signal and *direction* = signal-to-world) become differentiated.

Thus, Millikan acknowledges that animal signals are bidirectional, but linguistic utterances are not. I will now turn to a criticism of Millikan’s position, using two kinds of arguments: general arguments regarding the very notion of a linguistic signal in signal-information/response pairings, and pragmatic arguments regarding signal-information/response pairings.

### Some Difficulties with Millikan’s Position

The very structure of Millikan’s theory raises major difficulties and those difficulties are all linked, in one way or another, to the essential historicity of Millikan’s notion of signal, inherited from her notion of proper function. Basically, for pairings such as those that Millikan proposes as the origin of signals to occur, the signal-type, the information-type, and the response-type each have to be perennial and the repeated couplings between signals of that type, information of that type and responses of that type also have to be perennial.

This raises difficulties for the three main components of the pairings:

• Signals;
• Information;
• Responses.

I will examine them one after the other.

#### Signals

A first and major question is what a linguistic signal should be. Under Millikan’s broad definition of a signal, something is a signal if its proper function is to trigger a specific response in an audience, through information transfer.

Signals have to be units of communication, i.e., they have to transfer the information/produce the response in their own right. Basically, this means that they have not only to be semantic units, but also have to be communicative units (though the two normally coincide in holistic animal signals, as we shall see, they do not in language). Traditionally, it has been considered that language is *doubly articulated*^15^: on a rough and ready description, at the phonological level, phonemes are combined into meaningful words; at the syntactic level, words are combined into meaningful sentences. Clearly, phonemes, being semantically vacant, are not semantic units, and hence not signals. So, the first candidates for signals are words. On the face of it, they seem to be good candidates: they are perennial enough both in their forms and in their meanings^16^. The main problem with words is that, while they are semantic units, they are not communicative units. Though shouting “Fire!” may be a perfectly well-formed communicative act in some circumstances, most linguistic communicative acts do not correspond to isolated words. This leaves us with the sentence, understood as a utterance-type.

There is, however, a major problem with the notion that sentences are linguistic signals in the required sense. Couched as an argument:

**Lack of History Argument (Syntactic)**: Given linguistic creativity, sentences are fairly often one-off, that is, they lack the history necessary to the establishment (through signal-information pairing due to repeated correlations of signal and information) of a proper function.

To show why this is the case, I will now examine (and reject) an objection to the notion that language is characterized by linguistic creativity. This objection targets one of the core properties of language, i.e., discrete infinity.

It is to the effect that humans being finite cannot be said to produce an infinity of different sentences. This *Finitude* Argument has been formulated as follows by Li and Hombert (2002, p. 196): “Theoretically the number of possible sentences in English is indefinitely large because theoretically ‘the longest English sentence’ does not exist. If one chooses to describe English syntax or certain aspect of English syntax in terms of rewriting rules, one can claim that a recursive function is needed. However, one never conjoins or embeds an indefinitely large number of sentences in either spoken or written language. ‘Indefinitely large number of sentences’ or ‘infinitely long sentences’ are theoretical properties.” This seems to rests on a profound misunderstanding of both discrete infinity and recursion. To see it, an analogy with another system providing discrete infinity, i.e., mathematics, is useful. Saying that, because we do not (and could not, as finite beings) produce infinitely long sentences, discrete infinity and recursion are not relevant features of language is on a par with saying that, because we do not (and could not) count to infinity, discrete infinity and recursion are not relevant features of mathematics. The argument is, to say the least, mystifying. Arguably, recursion is needed to count up to any number greater than one, just as it is needed to produce any sentence with an embedding. Once you have the relevant recursive ability, you have the theoretical possibility of counting to infinity or to producing infinitely long sentences, and whether you do it or not is utterly irrelevant. Discrete infinity is a structural, not a behavioral property. Thus, human finitude is no argument against linguistic creativity.

More crucially, the argument is no answer to our worry regarding the absence of history for sentences. Even though each human, being a finite organism, cannot produce an infinity of different sentences with different contents, linguistic creativity *as a structural property of language* allows each human to produce sentences different from all those produced before, with contents different from all of those produced before. This being so, the fact that sentences may not have the necessary history to function as signals in pairs of signal-information/response remains a central problem. In sum, human finiteness is not an argument against linguistic creativity and is no answer to the absence of history for sentences.

This, then, is the first major problem for Millikan’s theory and it is, obviously, a syntactic argument. There are, however, further objections to her proposal and we will now turn to information.

#### Information

Regarding information, Millikan has concentrated on two main pragmatic phenomena, illocutionary force (Millikan, 1984, 2004, 2005) and implicatures of the scalar variety (Millikan, 2005). Beginning with the former, from 1984 on, her argument has been mainly based on the pairing between sentence forms (affirmative, interrogative, imperative, etc.) and the corresponding speech acts, covering both information and response. Leaving responses aside for further discussion later on, let us concentrate on information^17^. The “information” pairing is between sentence form and illocutionary act (or illocutionary force) and, as Millikan herself acknowledges (following Strawson, 1964), fairly often, an utterance can be linked to widely different illocutionary forces. Consider (3):

(3) Peter will come tomorrow.

Depending on the circumstances, this can indeed be interpreted as a promise, a menace, a warning or a prediction. Millikan proposes to get around this problem through a multiplicity of (proper) functions. As said above, the proper function of an entity is not what it actually does but why it has persisted through time. And even if it is not *always* reliably associated with that function, it is sufficient that it is associated with it *often enough*. Thus, the existence of occasional functions different from the proper function of a sentence is not a problem. Here, it is interesting to look at Millikan’s view of language change (which concerns the emergence of implicature readings). According to Millikan, if a linguistic form with a given proper function becomes associated often enough with another different function, this second function will become its new or additional proper function. In other words, the proper function of a linguistic item depends on the frequency with which this item is associated with this function and a linguistic signal can have several functions, proper or otherwise.

Let us look at an example:

(4) The pianist played *some* Mozart sonatas.

Notoriously, this utterance can be given two interpretations:

(5) The pianist played *at least some* ( = *some and maybe all*) Mozart sonatas. [*semantic* interpretation].
(6) The pianist played *only some* ( = *some and not all*) Mozart sonatas. [*pragmatic* interpretation].

According to Millikan, the initial proper function of (4) is to communicate (5). However, (4) is sometimes used to communicate (6) and, in time, this gives rise to a new function for (4). In addition to (5), (4) has also the function of communicating (6).

There is something mysterious about the process, however. How is it, if the proper function of (4) is to communicate (5), that, on the first occasion of its being used to communicate (6), the hearer will recognize that this is the case? Here, we turn to a first pragmatic argument:

**First Occasion Argument**: If meaning is established through repeated pairings, for such a pairing to take off, the meaning of a linguistic signal (or construction) has to be established on the occasion of its first production. A pragmatic inference will more often than not be necessary.

Note that the same argument applies to (3) above. Suppose that the initial function of (3) is to convey the illocutionary force of prediction. How does (3) acquire the additional functions of conveying the illocutionary forces of warning, menace of promise?

A final problem to do with first occasion arises for those signals who are associated with a given speaker meaning on a single occasion (one-off), as is clearly the case for some creative metaphors, such as^18^:

(7) “She smiled herself to an upgrade” (Adams, 1979).
(8) “We laughed our conversation to an end” (Hart, 1992).

In such cases, there is no way to recover the intended meaning through semantic compositionality, and pragmatic inferences to the speaker’s intentions are obviously necessary.

This is not the only difficulty, however. If a single linguistic signal can have several (proper) functions, this approach leads to widespread ambiguity in linguistic signals. And this suggests a second pragmatic argument:

**Ambiguity Argument**^19^: This approach supposes widespread ambiguity in linguistic signals. The resolution of that ambiguity will have to be done through pragmatic inferences.

Note, however, that what is central to Millikan’s view is not the absence of context-based pragmatic inference *per se*, but rather the absence of the *Gricean* kind of pragmatic inferences. Specifically what this means is that Millikan does not reject contextualism as such but that she rejects any brand of contextualism in which either the context includes psychological representations (e.g., speaker’s intentions or beliefs) or the interpretation process leads to psychological representations (e.g., *By X, the speaker meant Y*).

Here, it is interesting to go back to Millikan’s analysis of natural signs. As she notes, while natural signs do not have proper functions, they are nevertheless paired with types of information: smoke and the presence of a fire, clouds and future rain, etc. However, while natural signs are factive, they are not necessarily paired bi-univocally with the information they convey. Sometimes, two different natural signs with identical forms will be associated with two different informations depending on which environment each of them occurs in. Let us take an example. It so happens that identical tracks can be left by, e.g., a small bird and a small rodent. However, in wood A, there are only birds and no rodents, while in wood B, there are only rodents and no birds. Thus, natural signs with the same form will be read (factively) as corresponding to birds in wood A and to rodents in wood B. In other words, even natural signs can be context-dependent relative to the information they convey. If this is the case, why not apply the same solution (context-dependency) to sentences? Sentences would always be associated with context types, and utterance types would correspond not to sentences, but to couples of sentences and context-types. It is these composite utterance types that would be paired with proper functions, rather than sentences in isolation. And, obviously, such composite utterance types would make perfect sense as signals in signal-information/response pairs. Note that on such a view (which reflects Millikan’s see Millikan, 2004, Chap. 10), nothing like a Gricean “psychological” account is needed. The type of contexts concerned do not include any representation of the speaker’s intentions or beliefs, or indeed, of anyone’s mental states.

Let us now come back to example (3) above. As said before, a sentence such as *Peter will come tomorrow* may be understood as a promise, a menace, a warning or a prediction. Can we make sense of this in terms of utterance type, i.e., in terms of couples of sentences and (non-psychological) context types? In this specific case, it seems rather difficult to distinguish between these different illocutionary forces without appealing to mental states in both the speaker and the hearer. Presumably, leaving aside the fairly neutral speech act of prediction, what illocutionary force such an utterance will have will very much depend, not only on the speaker’s intention but also on what she knows, or believes she knows, about her hearer’s mental attitudes to Peter’s coming. The same reasoning applies to (4): whether it will be interpreted as (5) or (6) will depend at least in part on the intention the hearer attributes to the speaker.

In other words, the requirement that the context be non-psychological seems a gratuitous complication as far as linguistic communication is concerned, as distinguishing between different illocutionary forces will, more often than not, depend on the representation of the relevant attitudes in the speaker, the hearer or both. There is yet another worry, which again, goes back to the first occasion argument. Given that utterance types are themselves composite, being couples of sentences and context types, one can also ask how such couples come into existence, leading to a higher order first occasion problem. This problem is especially acute for linguistic communication, given decoupling, which allows speakers to speak of absent or non-existent objects, introducing a further difficulty as both the signal and its referent have to be present for any association process to operate.

Hence, neither the assumption of widespread ambiguity for sentences, nor the assumption of composite utterance types, leading to semantic inflation, can work given psychological parsimony. Basically, exchanging *semantic parsimony* + *psychological inflation*, as proposed by Grice, for *semantic inflation* + *psychological parsimony*, as proposed by Millikan, is not tenable. Whether one goes for semantic parsimony or for semantic inflation, one cannot escape psychological inflation. Thus, it does not seem that composite utterance types can play the role of signals in signal-information/response pairs either.

#### Responses

Let me now come to my third objection to Millikan, relative to the response type associated with the signal. Going back to Millikan’s central example, speech acts, the “information” pairing is between sentence form and illocutionary act, but the “response” pairing is between sentence form and perlocutionary act. Here, it is important to see why Millikan shares with Maynard Smith and Harper the view that it is not signals that have evolved, but rather signal–response pairs. This makes sense on an evolutionary view (be it biological or cultural) because, while conveying information does not as such make sense in evolutionary terms (information is a precious commodity, so why share it?), triggering responses in others, as long as these responses are advantageous to the signaler, makes perfect sense. So, on a view such as Millikan’s, according to which language is a communication system, it seems reasonable to see linguistic signals (whatever they are) as paired with responses rather than only with information.

Millikan’s main example is assertion, which, on the response side, is, according to her, paired with receiver’s belief. Obviously, not all assertions lead to receiver’s belief, but, as indicated above, for the pairing between assertion and receiver’s belief to be established (or, in other words, for receiver’s belief to be the proper function of assertion), it is sufficient that assertion be paired with belief often enough. Here, I want to discuss the appropriateness of belief as a receiver’s response in an evolutionary perspective.

On the face of it, it would seem that any receiver’s response in signal–response pairs should be detectable if the pairing is to have evolved^20^:

**Detectability of Response Argument**: for signal-response pairings to get off the ground, both the signal and the response must be detectable (respectively, by the receiver and by the signaler).

The problem with belief is not only that it is a mental state (and as such less easy to detect than a behavior or an action); it is in addition especially difficult to detect among mental states. While intentions are fairly often obvious from bodily preparation for action^21^, and emotions or feelings are detectable through facial expressions, belief seems to be wholly internal and not linked to any specific exteriorization^22^. One could argue of course that, given a belief with a certain content in her hearer, the speaker can detect its presence through his behavior interpreted *via* Theory of Mind, i.e., *via* the attribution of mental states. This, however, not only seems uncertain (see below), it also is not clear whether Millikan would agree with such a development, which is tantamount to re-introducing a rather Gricean (psychological) factor in the evolution of communication. Thus, belief appears to be a fairly strange candidate for a response in signal–response pairings.

This, however, is only a first objection. A second, and potentially more decisive objection is that responses, on such a view, have to be advantageous to the signaler (or, in the case of language, to the speaker). But belief as such is not advantageous to the speaker. Rather it is the behavioral consequences of the receiver’s belief (his deciding “to act on his belief”, so to speak) that may be advantageous to her. But, how exactly a hearer will act on his belief will depend on a host of other things, including his other beliefs and his desires, which strongly underdetermines the behavioral consequences of his (speaker induced) belief. Let us suppose, for instance, that John wants to go, while Mary wants him to stay. Mary could say:

(9) It is raining.

While the belief that it is raining might indeed induce John to stay, it might equally well make him take his umbrella, phone for a taxi or do a number of other things, none of which is staying, and none of which is what Mary wishes him to do. In other words, even in such simple cases, hearer’s behavioral responses are far from being obvious and there is certainly no way to predict them with any degree of certainty. And linguistic communication is of course far from being limited to such simple circumstances. In other words:

**Underdetermination of Behavioral Response Argument**: In humans at least, the automaticity or even the frequency of a given response to a given linguistic signal is largely underdetermined, undermining the pairing of signals and responses.

So Millikan’s choice of example, associating a linguistic signal (assertion) with a response that is a mental state (belief) can be explained through the fact that human action is not so automatic that it can be reliably associated with signals, barring imperatives in such strongly authoritative circumstances that the hearer has no choice but to comply. This, however, has two fairly negative consequences for her view of the evolution of linguistic communication: first, mental states are not the most detectable of responses, which raises a major difficulty for a signal-response pairing account such as hers (Detectability of Response Argument); second, mental states are additionally only indirectly advantageous to the speaker: they can only be advantageous to her if they lead her hearer to a behavior that she wants him to perform, but this is uncertain in most cases (Underdetermination of Response Argument).

Thus, Millikan’s endeavor to “de-psychologize” language and range it among all other animal communication systems fails. We will now turn to Scott-Phillips’s (2015) highly different view of language as a communication system.

## Language as a Communication System under the Ostensive Model

*Ostensive communication*, a notion that Scott-Phillips borrows from Sperber and Wilson (1995), corresponds to the view that human communication is intimately linked to the crucial notion of *relevance*. Relevance is a minimax notion and the communicative version of relevance goes as follows:

**Relevance**: An utterance is relevant to the extent that:

• It is less costly to interpret;
• It produces cognitive effects.

The cognitive effects produced by the interpretation of an utterance can be of three sorts: strengthening or weakening the conviction with which previous assumptions are entertained; deleting a previous assumption that is contradicted by the new information obtained (depending on the confidence the hearer places in the speaker); producing new assumptions. The *Communicative Principle of Relevance*^23^ says:

Every utterance carries the guarantee of its own optimal relevance.

*Optimal relevance* is achieved when the cognitive effects of an utterance balance its interpretive costs. The reason why utterances carry the guarantee of their own optimal relevance is because any utterance is an instance of *ostensive-inferential communication*. A behavior is an act of ostensive-inferential communication in as much as it makes it obvious to the receiver that the signaler has produced it with a communicative intention—this is the *ostension* part—and it is produced as evidence to be used in the inferential process through which the receiver will recover the signaler’s informative intention (i.e., the content she intended to communicate)—this is the *inference* part. Thus, an act of ostensive communication guarantees that it is worthwhile for the hearer to pay attention to it. Hence, by putting ostensive-inferential communication at the heart, not only of linguistic communication, but, as we shall now see, of language evolution, Scott-Phillips is taking a position which is the opposite of Millikan’s relative to language. Millikan’s rejection of inferential pragmatics and insistence on signal–response pairings makes her analysis unable to deal with the semantic underdetermination that is characteristic of linguistic communication. Scott-Phillips’ proposal can deal with it. But, as we shall see, it does more than that: his proposal basically reverses the problem.

At the center of Scott-Phillips’ view is a distinction between *natural codes* (which correspond to what Millikan describes) and *conventional codes* (which do not). The originality of Scott-Phillips’ proposal is to see ostensive communication (a short-hand for ostensive-inferential communication) not as a way of solving the problem of the semantic underdetermination of the conventional linguistic code (which would thus still be the basic root of linguistic communication), but as itself the root of human, including linguistic, communication, the conventional codes constituting language as a system being added to give human communication more expressive power. In other words (Scott-Phillips, 2015, p. 577), “there is a qualitative difference between the codes used in the code model, and the linguistic code. Put simply, one makes a type of communication possible, the other makes a different type of communication expressively powerful.” Conventional codes are ubiquitous in language, being found at the phonological, lexical, syntactic and even pragmatic (e.g., politeness conventions) levels. Scott-Phillips (2015, pp. 628–629) concludes: “This view of a language as a set of conventional codes that augments ostensive communication recognizes both the pragmatic foundations of linguistic behavior, and the importance and nature of the conventions that make languages different to other, simpler cases of ostensive-inferential communication, such as points, non-linguistic vocalizations, nods of the head, and so on.”

So, to sum up, on Scott-Philipps’ view, language is indeed a communication system, but it is a communication system entirely discontinuous with most if not all animal communication systems as it has evolved in the wake of abilities for ostensive communication that themselves depend on the previous evolution of a sophisticated Theory of Mind, developed on the basis of pre-existing primate abilities in social cognition, but outstripping them by far. Language itself is a collection of conventional codes, which greatly enhance the expressive power of ostensive communication, but which, nevertheless, are still in need of pragmatic inferencing, as they are, more often than not, semantically underdetermined relative to speaker’s meaning.

There is no doubt that Scott-Phillips’ proposal differs in many ways from Millikan’s. There is, however, one point on which they seem to meet. It is highly difficult, from Scott-Phillips’ presentation to see where exactly his conventional codes would differ from constructions, and, as we have seen, Millikan is also something of a constructivist. What is more, Scott-Philipps adopts a few other constructivist tenets. For instance in his fifth chapter, he rejects the Chomskyan notion of Universal Grammar^24^, which he sees as unnecessary. He also rejects the idea that recursion is a central factor in syntax and in linguistic creativity, though he seems to accept linguistic creativity in as much as he claims that linguistic communication is unlimited in the number of different contents language may be used to communicate.

This is not the only aspect in which Scott-Phillips’ theory meets Millikan’s. Another important meeting point between the two accounts is the notion of a signal-response pair as the basic communicative unit. Basically, Scott-Phillips distinguishes between *signals, cues, coercion, accidents*, by whether or not the behavior is designed to give rise to (designed) responses. In the case of a signal (the only communicative unit), the signal is designed to trigger the designed response (very much in keeping with Maynard Smith and Harper’s definition, see Language as a Communication System under the Code Model). The cue is not designed to trigger the response, though the response is designed as a response to that type of cue. In coercion the action is designed to trigger the response, but the response is not designed as a response to that type of action. And finally in an accident, neither the accident nor the response are designed relative to one another.

Given these two important points of agreement between Millikan’s and Scott-Phillips’ views, it makes sense to ask whether Scott-Phillips’ proposal falls foul of the objections raised above against Millikan’s. Obviously, the pragmatic objections (First Occasion Argument and Ambiguity Argument) do not apply. But, as we shall see, both the Lack of History Argument and the Underdetermination of Behavioral Response Argument do apply to Scott-Phillips’ theory.

As discussed above, any theory that defines communicative units as the result of pairings between signals and information/responses *ipso facto* supposes perenniality in signal types, in information types, in response types and in the pairings that link them. Scott-Phillips differs from Millikan in acknowledging from the start that the information communicated by different utterances of a given sentence will differ from occasion to occasion, and he does not explain this through widespread ambiguity. He explains it through the deep semantic underdetermination of linguistic (conventional) codes. This deep underdetermination affects speaker’s meaning, and makes it necessary for the conventional codes to be supplemented by pragmatic inference. While on Scott-Phillips’ model, pragmatic inference is available, this nevertheless means that different utterances of the same sentence will not be repeatedly paired with the same information. This leads us to a *pragmatic* version of the Lack of History Argument:

**Lack of History Argument (Pragmatic)**: Given semantic underdetermination, the speaker meaning attributed to one utterance of a given sentence will often be one-off, that is, it will not necessarily be attributed to any other utterance of the same sentence. In other words, utterances lack the semantic stability necessary to the establishment of a conventional code.

Let us now turn to responses. The example Scott-Phillips gives of a signal is of a man pushing a woman down under the eyes of another colleague, who laughs in response^25^. The pushing was intended to be seen by the laughing colleague and thus it is a communicative signal designed to trigger as its designed response the laughter. While this example is certainly not susceptible to the Detectability of Response Argument (laughter being detectable), it nevertheless is susceptible to the Underdetermination of Behavioral Responses Argument. Rather obviously, the intended receiver might have remonstrated instead of laughing.

Thus, while Scott-Phillips offers an original and attractive theory, it falls foul of some of the same difficulties that plague Millikan’s. My diagnosis is that this can basically be explained by the fact that these difficulties come from what the basic proposition shared by the two views is: that language is a communication system.

## The Language-ready Brain

The proposition that language is a communication system imposes obvious constraints on the abilities that have to pre-exist for language to get off the ground. Unsurprisingly, given that communication is the epitome of a social phenomenon, these abilities are social. On the code model, the main constraint is *honesty* (see Animal Communication Systems)—and this is all the more important in language, given the opportunities for cheating that decoupling offers. This has led to the view that *altruism*, as a phylogenetic pro-social tendency, is a prerequisite for human linguistic communication and for language evolution. On the ostensive model, linguistic communication and language evolution basically depend on the preexistence of a Theory of Mind of some kind. However, as we have just seen, the notion that language is a communication system in the strong sense that it evolved *for* communication is implausible in view of the difficulties it meets with. One fairly obvious suggestion to account for its use in human communication is that it originally evolved for entirely different purposes and was then exapted (Gould and Vrba, 1982) for communication. Determining what those purposes were is a prerequisite for determining which pre-existing abilities should comprise the language-ready brain.

Here, recall the two questions listed in Section “Introduction”, and more specifically the question of why humans—and only humans—need a system of communication that allows them to communicate a potential infinity of different contents. Communication is rife in nature, but language is unique. This immediately raises a further question: where does this infinity of different contents come from? As Millikan (2013) rightly notes, human cognitive sophistication is also unique. Thus, one potential answer to the question above is that a cognitively sophisticated species needs an appropriately sophisticated system of communication. What this means basically is that human intelligence, rather than human sociability, is the key to language. We can go one step further, however, following Fodor and Pylyshyn (2015), and note that thoughts and sentences share the same structural organization: just as sentences structurally compose words in a creative way, thoughts structurally compose concepts in a creative way. Language is creative, because thought is creative. Or, in Fodor and Pylyshyn (2015, p. 89) words, “That thoughts and sentences match up so nicely is part of why you can sometimes say what you think and vice versa.”

Hinzen (2013) goes farther and proposes that language is primarily an internal tool for thought and that syntax is the root of the semantic and propositional organization of thought in humans, and hence of the specificity of human thought, compared with non-human animal thought. This, it should be clear, also answers the second question raised in Section “Introduction”, i.e., why does linguistic communication allows decoupling which clearly facilitates cheating and deceiving? On a view in which language evolved for thought, discrete infinity, semanticity and decoupling are not structural features specific to linguistic communication, they are structural features specific to thought *and in no way dependent on whether language is externalized for communication* or not. Note that discrete infinity and decoupling, which are obvious embarrassments for a theory of language as a communicative system in the strong sense, raise no problem for a theory of thought: obviously, discrete infinity and decoupling are ways of exponentially increase thought production, while the question of honesty does not arise for thought. So, basically, all of this comes to the suggestion that language did not evolve *for* communication, it evolved *for* thought (as advocated by Chomsky: see, Chomsky, 2014). It allows us to construct what medieval philosophers (Panaccio, 1999) called *complex concepts*, propositions, judgments, etc. This is essentially Fodor’s *Language of Thought Hypothesis* (Fodor, 1975, 2008). Language was then externalized for communication, and its externalized version inherited its core combination of properties.

While this explains why language is such an exotic communication system^26^, it does not, in and off itself, explain why such a sophisticated system of thought is unique to humans: why did humans—and only humans—need such a sophisticated system of thought? Another human specificity is the richness of the human conceptual system. While some core conceptual mechanisms (Carey, 2009) may be shared with other species (notably with great apes, see Gómez, 2004), the extent of the human conceptual system is unique. This difference is obvious from the very limited size of the vocabulary acquired by animals engaged in language research programs (≈300 words) as compared to the size of human vocabularies (300 words at 3-year-old, 6,000 at 6-year-old, and around 200,000 at 18-year-old; for animals’ lexicon, see Anderson, 2004; for human lexicons at different ages, Bloom, 2000). While some of the difference may be due to externalized language itself, it is highly unlikely that this is the only explanation. Indeed, other considerations militate against an identity between the human conceptual system and non-human animal conceptual systems, including those of other primates. While monkeys can learn to visually categorize images (Fabre-Thorpe, 2003), they usually do so after intensive training (involving thousands of trials), by contrast with young children who learn new concepts (and the corresponding words) instantaneously (Bloom, 2000; Waxman, 2004). Apart from any reservation, one might have to consider visual categorization as a proof of concept possession, this hints at highly different mechanisms of conceptualization. Finally, though Orangutans may be an exception (Vonk and MacDonald, 2004), other great ape species, though able of categorical discrimination at different levels of abstraction, present a highly different profile from what is found in humans: the intermediate or basic level (roughly corresponding to the level of the species), which is by far the most easily accessed in humans, is the most difficult for them (it is the level at which they fail to transfer learned categories: see Vonk and MacDonald, 2002). Thus, all in all, there are good reasons to doubt that conceptualization follows the same path in humans and in non-human animals.

Here, the hypothesis is that different mechanisms operate in human conceptualization, explaining why humans have conceptual repertories so much wider than other species do. Having a huge conceptual system, however, can only be useful if the concepts can be assembled into complex concepts or propositions (thoughts). While association can bind concepts together (and does in both human and non-human animals), it power is limited: at most, it could lead to sequences of concepts. On the other hand, syntax allows structured and compositional mental representations to emerge (see Hinzen, 2013; Fodor and Pylyshyn, 2015 for more detailed arguments). The suggestion is thus that syntax emerged at the mental level to organize concepts into (propositional) thoughts.

The obvious question is what led to the emergence of such different mechanisms of conceptualization in humans. Here, it is hard to avoid speculation, but, as argued by Boeckx and Benítez-Burraco (2014a,b) and Benítez-Burraco and Boeckx, 2015, there are important differences between modern humans and the Neanderthal/Denisovan branch, the main one being the globularity of the modern human skull compared to the elongated shape of the Neanderthal/Denisovan skull. Boeckx and Benitez-Burraco hypothesize that this change in shape corresponds to major brain reorganization leading to greater cerebral connectivity. Additionally, they point out that this change is not so much due to the enlargement of the frontal lobes as to the expansion and reorganization of parietal areas. Now, one of the peculiarities of the Neanderthal/Denisovan skull is the so-called Neanderthal bun, a bump on the occipital part of the skull, corresponding to the primary visual cortex (V5, Brodmann area 17). While there is clearly more to conceptualization than perception, it is hard not to link the change in human conceptualization to the specificity of human perceptual preference for global processing of visual scenes (Navon, 1977; Kimschi et al., 2005) as opposed to non-human primate perceptual preference for local processing (Fagot and Deruelle, 1997; Fagot and Tomonaga, 1999; Fagot et al., 1999, 2001). It is not impossible that the reorganization assumed by Boeckx and Benítez-Burraco also concerned the occipital area with capital consequences on visual preferences in modern humans, leading to improved conceptualization.

## Conclusion

While most of this paper has been dedicated to show that language is not a communication system in the strong sense (i.e., it did not evolve *for* communication) and to outline an alternative cognitive account and its consequences for the language-ready brain, I do not want to close it without saying a word about the externalization of a pre-existing language for communication. While I criticized Scott-Phillips’ (2015) account above, I nonetheless think that it makes a lot of sense as an account of the *externalization* of language. As noted above (see Language as a Communication System under the Ostensive Model), Scott-Phillips, following Sperber and Wilson (1995), sees language as a sophisticated brand of ostensive communication and proposes that a less sophisticated and wholly unconventionalized brand of ostensive communication preceded the formation of linguistic conventions. As he notes, all ostensive communication rests on mind-reading abilities. While he is content to suppose that such abilities somehow derived from previous primate social abilities, this is unlikely for a number of reason, the main one being that primates seem pretty restricted in that area. At best, chimpanzees may be able of recognizing intentions (and even that is in dispute: for a general presentation, see Lurz, 2011). Scott-Phillips gives no reason why mind-reading abilities would make such a jump in humans. The hallmark of human mind reading is that, in Dennett’s (1987) words, it involves higher-order intentions (e.g., *Peter believes that Mary believes that p*), in other words, metarepresentations. Now metarepresentation crucially depends on recursion as the representations involved are structurally recursive. Under the scenario I propose, the development of recursive syntax in the Language of Thought allowed humans to develop mind-reading abilities far in excess of anything to be found in non-human species. This allowed humans to indulge in ostensive communication, leading to linguistic conventions, roughly along the lines indicated by Scott-Phillips. Note, in addition, that under this revised scenario, acquiring words means matching words to pre-existing concepts (as largely recognized in the lexical acquisition literature, Bloom, 2000). This largely dispels the problem described in the semantic version of the Lack of History Argument I opposed to Scott-Phillips’ view (see Language as a Communication System under the Ostensive Model). While speaker’s meaning has to be stable on the view that language evolved as a communication system (and clearly is not), sentence meaning stability is quite enough to ensure the establishment and learning of lexical conventions on the view that language is a communication system only in a weak sense. This is because language as a communication system in the weak sense can piggyback on the pre-existing conceptual system and Language of Thought, that, as argued by Hinzen (2013), fixes referential and propositional meaning.

## Conflict of Interest Statement

The author declares that the research was conducted in the absence of any commercial or financial relationships that could be construed as a potential conflict of interest.

## Footnotes

1 Számado and Szathmáry (2006) list eleven different scenarios (gossip, grooming, group bonding/ritual, hunting, language as a mental tool, pair bonding, motherese, sexual selection, song, status for information, and tool making), only one of which—language as a mental tool—is clearly and unquestionably non-social.

2 Basically, the two questions above subsume the four questions proposed in Számado and Szathmáry (2006).

3 Generally considered to have its origins in Shannon and Weaver’s (1949) theory of information.

4 Though for a dissenting view, see Owren et al. (2010) and Section “Animal Communication Systems”.

5 I leave a more complete presentation of an ostensive system of communication to Section“Language as a Communication System under the Ostensive Model” below, where Scott-Phillips’ proposal will be discussed.

6 Cheney and Seyfarth’s (1990) classical analysis of vervet monkeys’ alarm calls is an example of that strategy.

7 This is where the investigation into animal communication systems meets with the code model of communication.

8 Given their greater biological investment in reproduction in the vertebrate and even more in the mammal species, females are generally the “choosy” sex.

9 Interestingly, in their paper, Owren et al. (2010) strongly suggest that language and animal communication are entirely disjoint phenomena, a view with which I concur.

10 Initiated by Dretske (1981).

11 Which is where she parts ways with Lewis, 1969/2002 account of convention.

12 Again, though she borrows the term *coordination problem* from Lewis, 1969/2002, there is very little left of Lewis’ account of convention in Millikan’s theory. I will not discuss this here, as it is hardly central to my main purpose.

13 Subject to the same strictures as mentioned by Maynard Smith and Harper (2003): while the response must be advantageous for the sender, it must not be generally detrimental to the receiver, otherwise selection would eliminate receptivity to it. This led Maynard Smith and Harper to the (correct) conclusion that, on such an account, the evolution of signals is bound by honesty constraints.

14 There is evidence that some monkey species occasionally combine two calls to produce a modified meaning (Zuberbühler, 2002). These fairly limited phenomena are still poorly understood (for an intriguing pragmatic account, see Schlenker et al., 2014), but they hardly challenge the huge difference in compositionality between animal communication system and language (the only example of fairly sophisticated combinatoriality is birdsong, which, however, is not semantically compositional).

15 Anderson (2013) rightly points out that this is not, strictly speaking, correct, given that there is a third articulation at the morphological level. I will ignore this complication here.

16 Obviously, words change both in acoustic form and meaning with time. But while this may be a relatively quick process (taking at most decades rather than centuries or millennia), words still are stable enough to qualify as signals.

17 Setting aside both the evolutionary side of Millikan’s proposal as well as the pairings between signal and response, it is clear that Millikan’s view of the pairings between signals and information has much in common with contemporary constructivist approaches to language acquisition in linguistics (Goldberg, 2006). I will not discuss constructivism as such here for reasons of space. Note however that *mutatis mutandis*, the pragmatic arguments against Millikan’s account also apply to constructivism.

18 Strangely, these are given by Goldberg (2006, p. 6) as examples of constructions, that is as examples of repeated pairings between forms and functions.

19 This argument was first opposed to Millikan’s view (though not under that name) by Origgi and Sperber (2000).

20 No association is possible otherwise.

21 It seems indeed to be the mental state that most animals or young children detect fairly easily, though perhaps in less mental terms such as *goal* or *purpose*.

22 This may be because belief is phenomenologically vacant: there is nothing it is like to believe something outside of religious or quasi-religious (e.g., political, esthetic, and ethical) belief.

23 There is also a Cognitive Principle of Relevance, which we will ignore here.

24 Though Scott-Phillips acknowledges that there may well be linguistic universals, he proposes to explain them through Cultural Attraction Theory, not Universal Grammar.

25 He rightly notes that under his view one and the same behavioral token could be at one a signal, a cue, coercion and an accident depending on who observes it.

26 Because it is discontinuous from all other animal communication systems not merely by being the only ostensive communication system (Scott-Phillips, 2015, and see Language as a Communication System under the Ostensive Model), but also by *not* having evolved *for* communication in the first place.
